# Minimum effective volume of 0.2% ropivacaine for ultrasound-guided axillary brachial plexus block in preschool-age children

**DOI:** 10.1038/s41598-021-96582-3

**Published:** 2021-08-20

**Authors:** Liang Chen, Yang Shen, Shuangmei Liu, Yanyan Cao

**Affiliations:** 1 Department of Anesthesiology, Shengjing Hospital of China Medical University, No.36 Sanhao Street, Heping District, Shenyang, 110004 Liaoning Province China; 2 Department of Emergency Medicine, Shengjing Hospital of China Medical University, No.36 Sanhao Street, Heping District, Shenyang, 110004 Liaoning Province China

**Keywords:** Paediatric research, Peripheral nervous system

## Abstract

Ultrasound-guided axillary brachial plexus block is increasingly used in preschool-age patients. However, the minimum effective volume of local anaesthetics has not been determined. With ethical committee approval and written informed consent from the guardians of all paediatric patients, we studied 27 consecutive patients aged 3 to 6 years who were scheduled for hand surgery. After general anaesthesia, eligible patients received a set volume of ultrasound-guided axillary brachial plexus block. We determined the volume of 0.2% ropivacaine for consecutive patients from the preceding patient’s outcome. The initial volume was 0.4 ml/kg. The testing interval was set at 0.05 ml/kg, and the lowest volume was 0.1 ml/kg. The following conditions were defined as a successful block: no heart rate changes, body movement, or ventilatory disorders during the operation; no use of fentanyl in the PACU; and a postoperative sensory block score < 3. The sequences of positive and negative blocks in consecutive patients were recorded. Using probit regression analysis, the 50% effective volume was 0.185 ml/kg (95% CI 0.123–0.234), and the 95% effective volume was 0.280 ml/kg (95% CI 0.232–0.593). EV50 and EV95 values of 0.2% ropivacaine for ultrasound-guided axillary brachial plexus block were 0.185 ml/kg and 0.280 ml/kg, respectively.

## Introduction

Axillary brachial plexus block (ABPB) is a common anaesthesia method in upper limb surgery and, compared to other brachial plexus block approaches, presents almost no risk of phrenic nerve block or pneumothorax. However, it uses large amounts of local anaesthetics, increasing the risk of local anaesthetic toxicity.

In the past, nerve blocks were rarely used in children because children have poor compliance during puncture, and their body surface signs are less obvious. With the widespread use of ultrasound technology^1–3^, nerve blocks are now widely used in paediatric surgery and can reduce the amount of opioids required for general anaesthesia or as analgesics after surgery. However, the minimum effective volume (MEV) of ropivacaine required for ultrasound-guided brachial plexus nerve block has not been determined in children^4^. The volume of anaesthetic is an important factor in determining nerve block efficacy. There are four main nerves in the axillary brachial plexus: the ulnar nerve, the radial nerve, the median nerve, and the musculocutaneous nerve. In adults, four-injection (blocking all four nerves) and double-injection (musculocutaneous nerve and axillary artery sheath) techniques are used^5,6^. Due to the small volume of anaesthetics used in paediatric patients, we used a single point injection for ABPB. We designed this prospective sequential trial to determine the minimum effective volumes (EV50 and EV95) of 0.2% ropivacaine.

## Materials and methods

This study was conducted at Shengjing Hospital from March 2021 to May 2021 and approved by the Institutional Review Board of Shengjing Hospital of China Medical University, Shenyang, China (approval number: 2021PS443K, approval date: 10/03/2021). Letters of informed consent signed by the guardians of all paediatric patients were obtained. This study was conducted in accordance with the principles of the Declaration of Helsinki. The trial was registered at the Chinese Clinical Trial Registry (Registration Number: ChiCTR2100044656, Registration Date: 25/03/2021).

The inclusion criteria were as follows: preschool-age children aged 3–6 years old whose surgical site was the hand. The exclusion criteria were as follows: patients with coagulopathy, anticoagulant therapy, allergy to local anaesthetic, infection at the puncture site, nerve injury, sensory impairment in the hand, intellectual disability, and those who required surgery in both hands.

The Dixon “up-and-down” sequential allocation methodology was used in our experimental design^7^. Based on clinical experience, the initial volume of injection was set to 0.4 ml/kg 0.2% ropivacaine with a gradient of 0.05 ml/kg. The minimum volume was set to 0.1 ml/kg instead of 0 ml/kg due to ethical considerations. As child development varies widely and children are prone to overweight status, obesity, and other conditions, the standardized weights of the patients were obtained from their heights. The standard weight we use comes from *Reference standard for growth and development of children under 7 years old in China.* The standardized weights were compared with the actual weights of the children, and the lower value was used as the weight for the purpose of calculating the total amount of local anaesthetic.

The anaesthesia plan was to obtain venous access to the upper limb on the non-operated side as soon as the child entered the operating room. Blood pressure, pulse oximetry, electrocardiography, and body temperature were monitored. Patients were given oxygen at 3 L/min, 0.05 mg/kg intravenous midazolam, 2.5–3 mg/kg propofol, and 2–4 mg/kg/h propofol infusion for maintenance. After the patient lost consciousness and the eyelash reflex disappeared for 1 min, a laryngeal mask was fitted, mixed-air oxygen with an oxygen concentration of 30% was administered, and mechanical ventilation or spontaneous breathing was selected depending on the situation.

**Figure 1:**
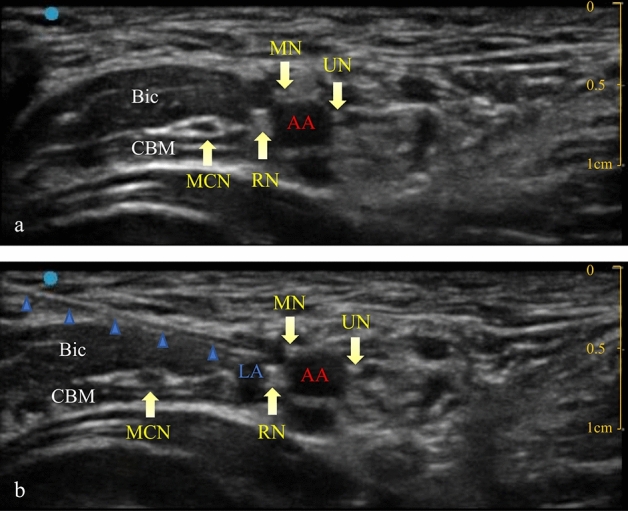
Ultrasound image preinjection (**a**) and ultrasound image postinjection (**b**). needle. AA: axillary artery; LA: local anaesthetics; MCN: musculocutaneous nerve; RN: radial nerve; MN: median nerve; UN: ulnar nerve; Bic: biceps brachii muscle; CBM: coracobrachialis muscle.

An ultrasound guided ABPB was then performed. The patient was placed in the supine position with the upper limb abducted on the surgical side. The elbow joint was flexed at 90°, exposing the armpit. A bedside ultrasound instrument (Venue 50, GE Medical Systems (China) Co., Ltd) was used with a high-frequency linear array probe (L8-18i, GE Medical Systems (China) Co., Ltd) in nerve block mode. The ultrasound probe was placed in the armpit, and the axillary artery was scanned along its short axis. The axillary artery was centred on the screen, and the locations of the three nerves around the axillary artery were determined. The median nerve is usually located at the 9–12 o’clock position of the axillary artery, the ulnar nerve is usually located at the 2 o’clock position, and the radial nerve is usually located at the 5 o’clock position^8^. Local anaesthetics were injected near the nerves innervating the skin of the incision. If the surgical area extended beyond the area innervated by a nerve, a single-point injection was still performed, and the anaesthesiologist selected the nerve to be injected. The needle was inserted from the outside of the probe, and the needle tip was placed near the axillary artery and the target nerve. A small amount of saline (not more than 0.5 ml) was injected to ensure that the needle tip was in an ideal position. Next, a set volume of 0.2% ropivacaine was injected. The ultrasound images before and after injection are shown in Fig. 1.

Surgery was performed 30 min after the block was completed. During this time, if the patient had ventilatory difficulty with the laryngeal mask, such as leakage above 2 ml/kg, tidal volumes below 6 ml/kg, ETCO2 above 45 mmHg, peak airway pressure above 20 cm H2O or the presence of audible stridor, 0.05 mg/kg cisatracurium was administered. If the ventilation problem was still unresolved, the patient was switched to tracheal intubation. These patients were excluded from the study. Surgical preparations were also performed during this time. A tourniquet was placed on the upper arm on the side to be operated upon, and a sterile drape was placed. Thirty minutes after the block was completed, the tourniquet on the upper arm on the side to be operated upon was pressurized to 20 kPa. The average heart rate 1 min before skin incision was considered the baseline heart rate. If the heart rate increased by over 20% above the baseline value, the patient exhibited obvious physical movement, or the patient experienced ventilatory difficulty after skin incision, 0.002 mg/kg fentanyl and 1 mg/kg propofol were administered, and the operation was continued after 2 min. For ventilatory disorders that could not be improved by fentanyl and propofol administration, 0.05 mg/kg cisatracurium was administered, and mechanical ventilation was used. After the operation, the propofol pump was stopped, the laryngeal mask was removed after the patient achieved good spontaneous breathing, and the patient was sent to the postanesthesia care unit (PACU). The time at which the block operation was completed and the times at which the operation was started and ended were recorded.

The Face, Legs, Activity, Cry, Consolability (FLACC) pain scale was used to score the pain of the patient in the PACU^9^. For those with a score of ≥ 4, 0.001 mg/kg fentanyl was administered. The patient was returned to the ward when fully awake. The PACU entry and exit times were recorded.

An anaesthesiologist evaluated the efficacy of the sensory block of cold sensation in the patient’s hand 30 min after the patient was returned to the ward. Alcohol-soaked cotton balls were used to evaluate cold sensation in the hand areas innervated by the three aforementioned nerves. Compared to the non-operated hand, 0 was described as no sensation in the blocked hand, 1 was described as weak sensation in the blocked hand, and 2 was described as the same feeling in both hands. If the sensation in the innervated area could not be assessed, it was indicated as a missing sensory block score.

To evaluate the duration of analgesia, the family members of the children were asked to record the time when the child felt pain for the first time.

Finally, the preceding results were taken together to determine whether the ABPB was successful.

The following conditions were defined as a successful block: no heart rate changes, body movement, or ventilatory disorders during the operation, no use of fentanyl during the operation or in the PACU, and a postoperative sensory block score < 3. After a successful block in the patient, the volume for the next patient was reduced by 0.05 ml/kg.

Any of the following conditions was considered block failure: heart rate change of over 20%, significant physical movement, ventilatory disorders during the operation, use of fentanyl during the operation or in the PACU, or postoperative sensory block score of 3 or greater. After an unsuccessful block in the patient, the volume for the next patient was increased by 0.05 ml/kg.

If the patient exhibited no heart rate changes, body movement, or ventilatory disturbance during the operation and did not use fentanyl, but had a missing postoperative sensory block score, the volume for the next patient remained unchanged.

We selected two conditions for terminating the study. Based on previous non-probabilistic sequential administration and upper- and lower-dose discovery studies with similar binary results, we estimated that at least seven independent negative–positive up-and-down deflections were required to calculate the minimum effective anaesthetic volume (MEAV)50. In addition, it was known at this point that a local anaesthetic dose < 0.1 ml/kg has no clinical significance. Therefore, a volume of 0.1 ml/kg that resulted in successful block in five consecutive patients was considered the second end point^7,10^.

### Blinding method

All ABPB operations were completed by the same doctor (Doctor Chen), who did not participate in other aspects of the study. The doctors who performed anaesthesia, PACU monitoring, and postoperative evaluation did not know the volume of local anaesthetic used. Their observation results were passed to another specialized doctor (Yang Shen), who combined all of the results to confirm the volume of local anaesthetic for the next patient and informed Doctor Chen of the volume before the next patient was anaesthetized.

### Statistical analysis

SPSS 24 software (IBM Corp., Armonk, NY, USA) was used for statistical analysis. Data were expressed as an appropriate average (range). Continuous data are expressed as the mean ± standard deviation (x ± sd) or median (interquartile range), and count data are expressed as the number of cases or a percentage. Probit regression analysis was performed on local anaesthetic volume data to calculate the 50% (EV50) and 95% (EV95) effective volumes and their 95% confidence intervals (CIs). Pearson’s test was used to test the correlation between ropivacaine volume and block duration.

## Results

**Figure 2:**
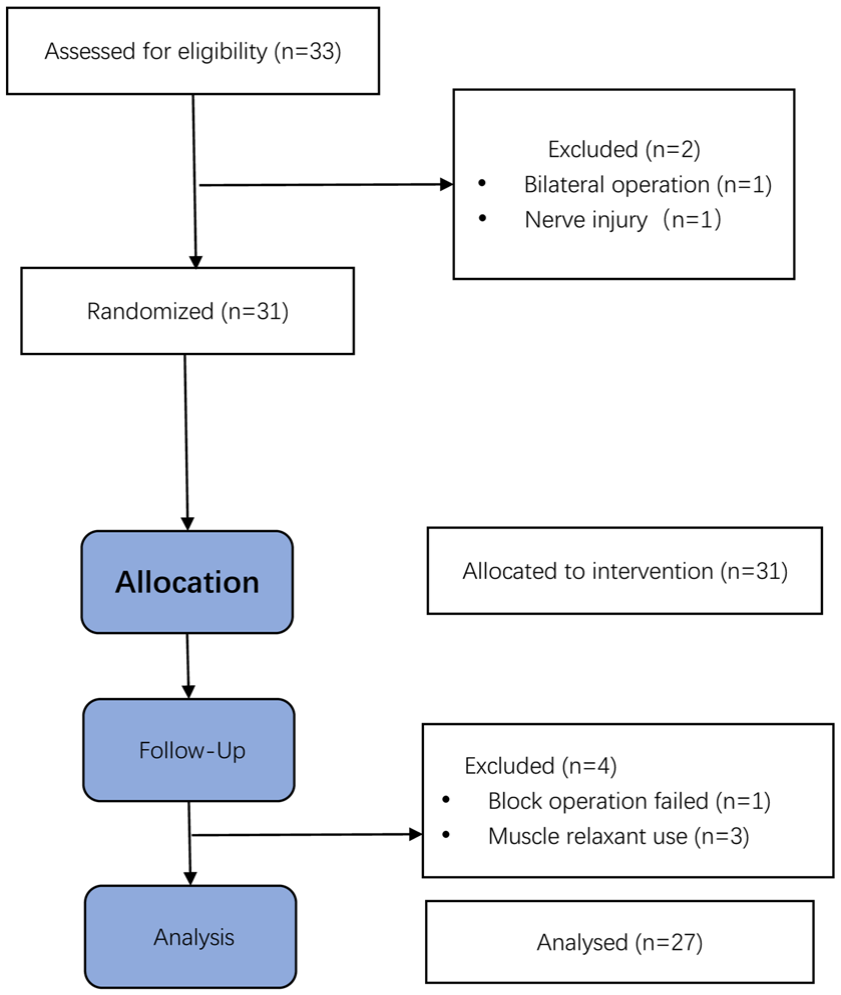
Flow diagram of the study.

From March 26, 2021, to May 10, 2021, a total of 33 children who met the inclusion criteria were enrolled in this study. A CONSORT flow diagram is shown in Fig. 2. Two children were excluded: one due to bilateral operation, and one due to possible nerve injury. Thirty-one children were included in the study. One child was excluded because of the failure of the axillary block operation, and three children were excluded by using muscle relaxants before the operation. Ultimately, 27 children completed the study and were analysed.

The characteristic data of the children are presented in Table 1. The volumes for three children were calculated according to the standard weights corresponding to height, and the data are also shown in Table 1.

**Table 1:** Patient characteristics.

	n = 27
Age (years)	4 [3–5] (3–6)
Sex: male/female	18/9
Height (cm)	107 [103–118] (95–131)
Weight (kg)	18 [16–21.5] (13–29)
Weight (kg) adjusted to standard weight*	17.5 [16–20.9] (13–28)*
ASA physical status (I/II)	25/2
Operation time (min)	50 [35–60] (20–90)
PACU time (min)	25 [20–30] (15–35)

Data are presented as the median [IQR] (range) or number (%).

ASA, American Society of Anaesthesiologists.

* Adjusted to standard weight according to height.

**Figure 3:**
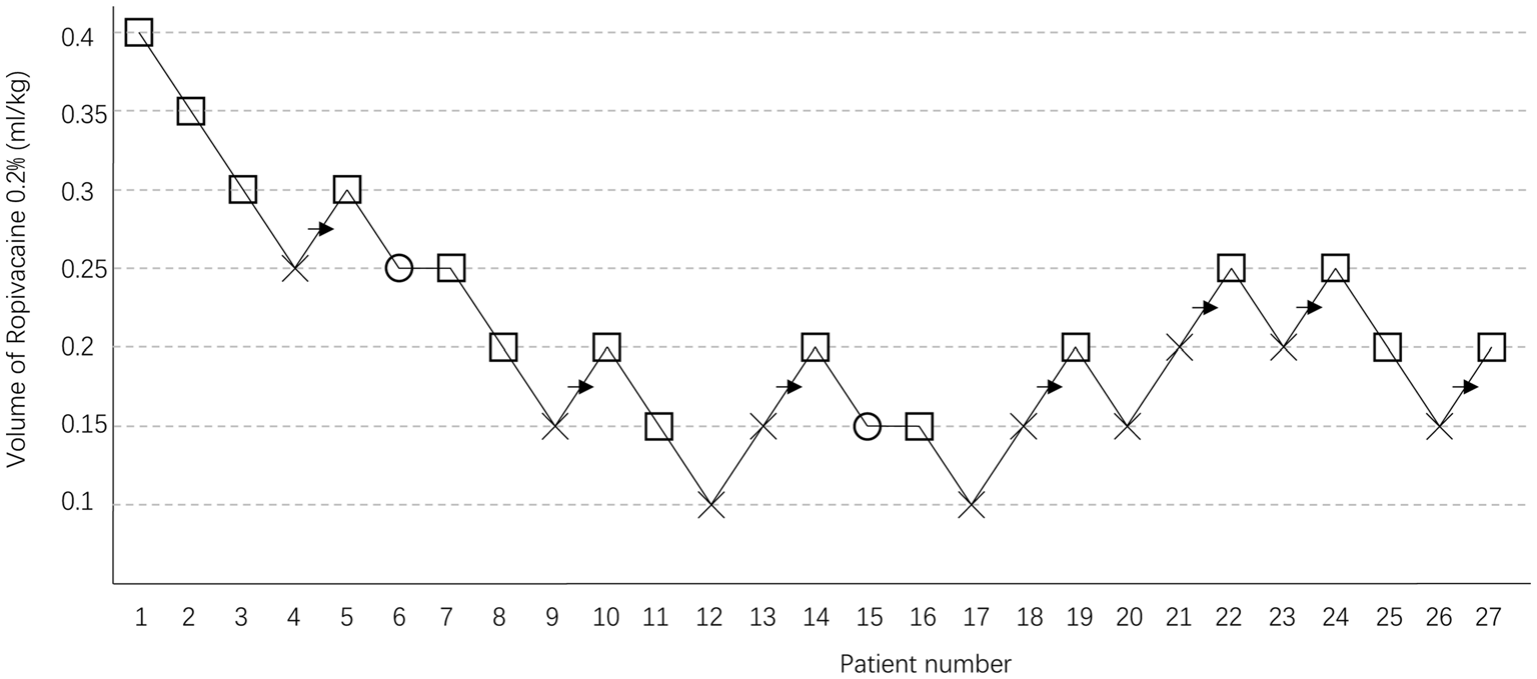
The up-and-down sequence of volumes of 0.2% ropivacaine required to produce an effective block. × failed block, □ effective block,○ loss of sensory block score, → midpoint of “failed-effective” pair.

The sequences of positive and negative blocks in consecutive patients are shown in Fig. 3. The EV50 and EV95 values were calculated using probit regression analysis. The EV50 and EV95 values of 0.2% ropivacaine for ultrasound-guided ABPB to produce an effective block were 0.185 ml/kg (95% CI 0.123–0.234) and 0.280 ml/kg (95% CI 0.232–0.593), respectively.

The number of successful cases in different ropivacaine volume groups is shown in Table 2. In all cases judged as successful blocks, the mean duration of analgesia was 289.0 ± 73.1 min. The duration of analgesia was not associated with the volume of 0.2% ropivacaine (Pearson r = -0.122, P = 0.665).

**Table 2:** The percentages of patients with effective blocks in each subgroup.

Ropivacaine volume in each subgroup (ml/kg)	Number	Success rate (effective/total)
0.10	n = 2	0% (0/2)
0.15	n = 7	29% (2/7)
0.20	n = 6	67% (4/6)
0.25	n = 5	80%(4/5)
0.30	n = 2	100% (2/2)
0.35	n = 1	100%(1/1)
0.40	n = 1	100%(1/1)

Data are presented as % (number).

Block failed in 10 patients. Five patients exhibited heart rate acceleration, body movement or ventilation disturbance after skin incision; four patients used fentanyl in the PACU; and the postoperative sensory block scores of five patients exceeded 3. Among the five patients whose postoperative sensory block scores exceeded 3, two patients had not used fentanyl before. The sensory block scores of two patients were missing because the hand was covered with plaster.

## Discussion

The present study revealed that the EV50 and EV95 values of 0.2% ropivacaine for ultrasound-guided ABPB in preschool-age children were 0.185 ml/kg and 0.280 ml/kg, respectively.

Nerve blocks are becoming increasingly common in paediatric surgery. Compound nerve blocks can reduce the demand for opioids. Compared to adults, children are more sensitive to opioids, and the large differences in the responses of different children to opioids result in a high incidence of postoperative opioid-associated alveolar hypoventilation^8,11^. Alveolar hypoventilation is the most serious side effect of opioids because it may cause hypoxia-related brain damage and death^12^. In children undergoing hand surgery, a successful brachial plexus block can reduce or even eliminate the use of opioids, thereby reducing potential risks while still ensuring adequate postoperative analgesia.

Determining the MEV of nerve block is difficult in paediatric patients because the efficacy of the nerve block cannot be accurately assessed. In our experimental design, midazolam and propofol were used for general anaesthesia before nerve block was performed, although these two anaesthetics exert no analgesic effects. Therefore, we were able to determine the efficacy of nerve block after the operation began. In theory, only propofol is needed for general anaesthesia, but midazolam was used during induction to prevent intraoperative awareness caused by insufficient analgesia due to failure of the nerve block. In the case of ventilation disturbance, we used a muscle relaxant and then switched to mechanical ventilation. However, body movements could not be judged in these patients; thus, they were excluded.

Two common techniques for ABPB are the perivascular (PV) and perineural (PN) techniques. Several studies on the PV technique found that complete block can be successfully achieved with one injection near the axillary artery, with a faster onset, a lower dosage, and less discomfort and pain^13–15^. The block method used in the trial was a combination of the PV and PN techniques. We selected a single injection of anaesthetic next to the axillary artery near the innervation site for the surgical incision to achieve ABPB. Under ultrasound guidance, the tip of the puncture needle was positioned as close as possible to the axillary artery while avoiding vascular damage or intravascular injection. Satisfactory injection efficacy was achieved if the anaesthetic solution was concentrated at the site of action, if the nerve was effectively surrounded by the solution, and if the axillary artery was compressed, indicating that the anaesthetic solution was accurately injected into the axillary artery sheath. Using a smaller volume can result in an effective block, and the local anaesthetic can even diffuse to the superior side, which may block the proximal end of the musculocutaneous nerve. The musculocutaneous nerve does not innervate the hand. Consequently, we did not administer anaesthetics to the musculocutaneous nerve.

In clinical practice, an important goal of musculocutaneous nerve block is to mitigate the reaction to tourniquet application. A previous study showed that the tourniquet pain onset time in patients undergoing ABPB was 73.0 (14.8) minutes^16^. The tourniquet undoubtedly produced some undesirable irritation, but this could be eliminated in a short period of time by continuous pumping of propofol without a tourniquet reaction. Our average surgical duration was 50.7 min, and the maximum duration was 80 min. Therefore, the tourniquet did not produce intraoperative irritation. However, care must still be taken to select an appropriate tourniquet pressure.

Follow-up was conducted on the patients after the surgical procedure. The FLACC pain scores of the patients were assessed in the PACU; scores ≥ 4 were generally considered moderate pain. We believed that incomplete postoperative metabolism of general anaesthetics, the anxiety of being separated from parents or from being in an unfamiliar environment, or plaster fixation after the operation can cause the patient not to feel pain, but not report a score of 0. However, these reasons were insufficient if the score reached 4, and it was considered that the patient had insufficient analgesia; thus, fentanyl was administered for analgesia. After the patient was fully awake and returned to the ward, the sensory block in the areas innervated by the three nerves was assessed further. The selected preschool-age children could accurately describe the changes in skin sensation in a waking state under normal circumstances. During the follow-up, we did not encounter any cases where the patients refused to or were unable to describe the sensations they experienced.

The physical development of children of different ages differs greatly. Even at the same age, the height and weight of different children can also differ. Therefore, paediatric MEV should not only be measured in ml; instead, ml/kg should be used to achieve individualized dosage. In addition, children are now more likely to be obese. When determining the weight of the child, we used the lower value of the actual weight and height corresponding to the standard weight. Therefore, the results of our study may be too high.

Previous trials have suggested that 0.2% ropivacaine can be used to achieve sensory block for over 9 h in adults^17^. We recorded the time of the first occurrence of postoperative pain in the patient, calculated the time for a patient who was judged to have a successful block, and determined that the duration of the block was 289.0 (73.1) min; the duration of block was not related to the volume of ropivacaine.

In our trial design, the volume for the next patient after a successful case was reduced, and there was no chance of the same volume being used on a patient. This was because of the strict conditions imposed for confirming successful cases. Therefore, for unsuccessful cases, the false positive rate may have increased, which may have also caused the values in our study results to be too high.

### Limitations

The present study had the following limitations: (1) to evaluate the postoperative efficacy of the block, we limited the age of the included patients to preschool age. In the initial trial design, we planned to recruit children aged 0–14 years. However, it was found that the age may have a great impact on the volume of ropivacaine. Therefore, in subsequent trial, the age of children was further subdivided. The method of the trial described in this manuscript is completely consistent with the trial registration. The MEV results in children of other ages will require further study; (2) in addition to volume, different local anaesthetics, different concentrations, and whether adjuvants are added also affect nerve block efficacy; and (3) we used a low dose of propofol to maintain anaesthesia in this trial with the objective of reducing the influence of propofol on the evaluation of the block effect. However, this may lead to exclusion of some patients with ventilatory difficulty. The results of the present study were obtained under the limited conditions of the study, and the dosages under other conditions require further study.

## Conclusions

In conclusion, this study demonstrated that the EV50 and EV95 values of 0.2% ropivacaine for ultrasound-guided ABPB in preschool-age children were 0.185 ml/kg and 0.280 ml/kg, respectively.

## Data Availability

The original data statistics table can be sent as an attachment upon request.
